# Impact of Tranexamic Acid for Hemostasis on Postoperative Complications in Pediatric Hypospadias: A Prospective Controlled Study

**DOI:** 10.7759/cureus.72116

**Published:** 2024-10-22

**Authors:** Shimeng Zhao, Can Qi, Pengyu Jia, Yan Hu, Ruifeng Gao, Hongchao Chai, Chaojun Xin, Yun Zhou

**Affiliations:** 1 Urology, Hebei Children's Hospital, Shijiazhuang, CHN; 2 Nursing, Hebei Children's Hospital, Shijiazhuang, CHN

**Keywords:** hemorrhage, hypospadias repair, intravenous tranexamic acid, ischemia-reperfusion injury, urethroplasty

## Abstract

Objective: This study aims to investigate the effects of intravenous tranexamic acid (TXA) hemostasis during pediatric hypospadias surgery on postoperative complications.

Materials and methods: A prospective randomized controlled study was conducted involving patients undergoing transverse preputial island flap urethroplasty (Duckett procedure) for hypospadias between January 2021 and February 2023, who were divided into the TXA group and the control group. Clinical parameters between the two groups were compared, and single-factor and logistic regression analyses were conducted to determine the impact of TXA application on postoperative complications in hypospadias surgery.

Results: A total of 124 hypospadias patients were followed up, with 64 in the TXA group and 60 in the control group. In the TXA group, intraoperative blood loss, electrotome usage, pre- to postoperative change in hemoglobin (ΔHB), and change in hematocrit were significantly lower compared to the control group (p＜0.05). Single-factor analysis of postoperative complications showed significant correlations with urethral defect length, electrotome usage, intraoperative blood loss, ΔHB, and bleeding complications after removing the penile bandage (p＜0.05). Multivariate logistic regression analysis indicated that TXA application (p=0.049), electrotome usage (p=0.003), intraoperative blood loss (p＜0.001), and ΔHB (p=0.001) were independent predictors of postoperative complications.

Conclusion: Intraoperative blood loss and pre- to postoperative change in hemoglobin are independent predictors of complications in the Duckett procedure for hypospadias surgery. Moreover, TXA can reduce blood loss and lower the risk of postoperative complications in patients undergoing the Duckett procedure.

## Introduction

Hypospadias is a common congenital developmental defect of the urinary system in children, characterized by an abnormal urethral opening that requires surgical treatment [1]. Transverse preputial island flap urethroplasty (Duckett procedure) is a commonly used surgical technique for hypospadias, involving the tubularization of a preputial island flap to reconstruct the urethra, which reported an overall complication rate of approximately 34% [2]. Controlling bleeding during hypospadias surgery not only optimizes the surgical field for clear anatomical visualization, which is beneficial for urethral reconstruction, but also reduces the risks of anesthesia, infection, and the need for perioperative blood transfusion [3].

Traditional methods to reduce bleeding during hypospadias surgery include penile tourniquets, vasoconstrictors like adrenaline, and electrotome, which temporarily reduce tissue perfusion. However, reperfusion may lead to the release of reactive oxygen species, inducing ischemia-reperfusion (I/R) injury and potentially affecting healing capabilities [4]. Tranexamic acid (TXA) is a widely used clinical antifibrinolytic agent that delays the degradation of fibrin clots, promotes hemostasis, and effectively reduces perioperative bleeding [5]. It also has beneficial effects in preventing inflammatory responses and mitigating I/R injury [6].

Currently, there are no reports on the use of TXA in pediatric hypospadias surgery. This study aims to investigate the impact of TXA on perioperative bleeding and the occurrence of complications in children undergoing the Duckett procedure for hypospadias through a prospective randomized controlled trial.

## Materials and methods

This study was conducted from January 2021 to February 2023, with data collection conducted prospectively and randomly. This study had obtained approval from the Ethics Committee of Hebei Children's Hospital (approval number: 202222-38), and all patients had signed informed consent forms. This study was registered on the China National Health Insurance Information Platform with the registration number MR-13-24-021975.

The inclusion criteria for the study were as follows: (1) primary hypospadias patients undergoing the Duckett procedure at our center; (2) patients under the age of 18; and (3) a follow-up period of at least 12 months. The exclusion criteria included: (1) a history of previous hypospadias or other penile surgeries; (2) the presence of cryptorchidism, penoscrotal transposition, disorders of sex development, or other congenital abnormalities of the genitourinary system; (3) coagulation disorders or a history of epilepsy; (4) previous injection or topical use of hormones on the penis; (5) an allergy to TXA; and (6) non-compliance with treatment or follow-up.

Patients were grouped using simple random sampling, and a surgical nurse flipped a coin upon admission to assign participants to either the TXA group (heads) or the control group (tails). The coin toss randomization process involved defining the study's inclusion and exclusion criteria to ensure that only eligible patients could participate. A trained surgical nurse conducted the randomization by performing the coin toss in a private setting upon patient admission. Immediately after the coin landed, the nurse recorded the results, documenting each patient’s unique identification code, group assignment (TXA or control), and the date and time of the toss. A standardized data collection form was used to ensure accuracy and traceability. To maintain the blinding of the study, measures were taken to ensure that patients, their families, and the operating physicians remained unaware of the group assignments. The TXA group received 10 mg/kg of TXA (with a maximum dose not exceeding 0.5 g per administration) dissolved in 100 ml of 5% glucose solution, administered intravenously 5-10 minutes before surgery and completed within 30 minutes. Postoperatively, the same dose of TXA was administered intravenously once daily for five days. The control group received 100 ml of 5% glucose solution intravenously 5-10 minutes before the skin incision, also completed within 30 minutes. The study was conducted using a double-blind method to ensure that neither the patients' families nor the operating physicians were aware of the group assignments.

Surgical methods

All surgeries were performed under general anesthesia. Hemostasis was achieved intraoperatively using the electrotome, and the number of hemostatic applications was recorded. No tourniquet or adrenaline was used for hemostasis. If the chordee was greater than 30° after the penile skin degloved and the urethral plate transected, dorsal plication (DP) was performed; along the tunica albuginea of the corpus cavernosum, Buck's fascia and dorsal vascular neurovascular bundle were dissected, and two sutures were placed to plicate the tunica albuginea dorsally, correcting the chordee and fully straightening the penis. A pedicled island flap of the dorsal skin, with a width of 1-1.2 cm and slightly longer than the length of the urethral defect, was mobilized. The flap was tubularized to form a new urethra, and the vascular pedicle was transferred to the ventral aspect of the penis. The distal end exited through a tunnel beneath the glans penis, and the proximal end angled to meet the original urethral meatus. The vascular pedicle covered the new urethra, and the penile skin was trimmed and sutured. Postoperatively, the penis wound was dressed with elastic bandages for five days. A urinary catheter was left in place for four weeks.

Observational indicators

Patient general information, hypospadias classification, operative duration, length of urethral defect, chordee >30°, use of DP, and the number of electrotome usages were collected. Blood loss during surgery was measured using a weighing method (weighing surgical gauze every 10 minutes with an electronic scale to minimize the impact of moisture evaporation on gauze weight). Preoperative and postoperative day 1 measurements included hemoglobin (HB), hematocrit (HCT), platelet count (PLT), prothrombin time (PT), international normalized ratio (INR), activated partial thromboplastin time (APTT), thrombin time (TT), fibrinogen (FIB), and D-dimer (DD). Differences between preoperative and postoperative values were calculated as ΔHB, ΔHCT, ΔPLT, ΔPT, ΔINR, ΔAPTT, ΔTT, ΔFIB, and ΔDD.

The classification of hypospadias was based on the position of the urethral meatus after correction of chordee: distal type (meatus located at the distal 1/3 of the penile shaft), midshaft type (meatus located at the midshaft 1/3 of the penile shaft), and proximal type (meatus located at the proximal 1/3 of the penile shaft).

Assessment of bleeding occurred five days postoperatively after removing the bandage from the penis, categorized as mild, moderate, or severe. We defined mild bleeding as minimal oozing from the incision that resolves spontaneously; moderate bleeding requires the reapplication of elastic bandages to achieve hemostasis; and severe bleeding necessitates suturing to control the hemorrhage.

**Figure 1:**
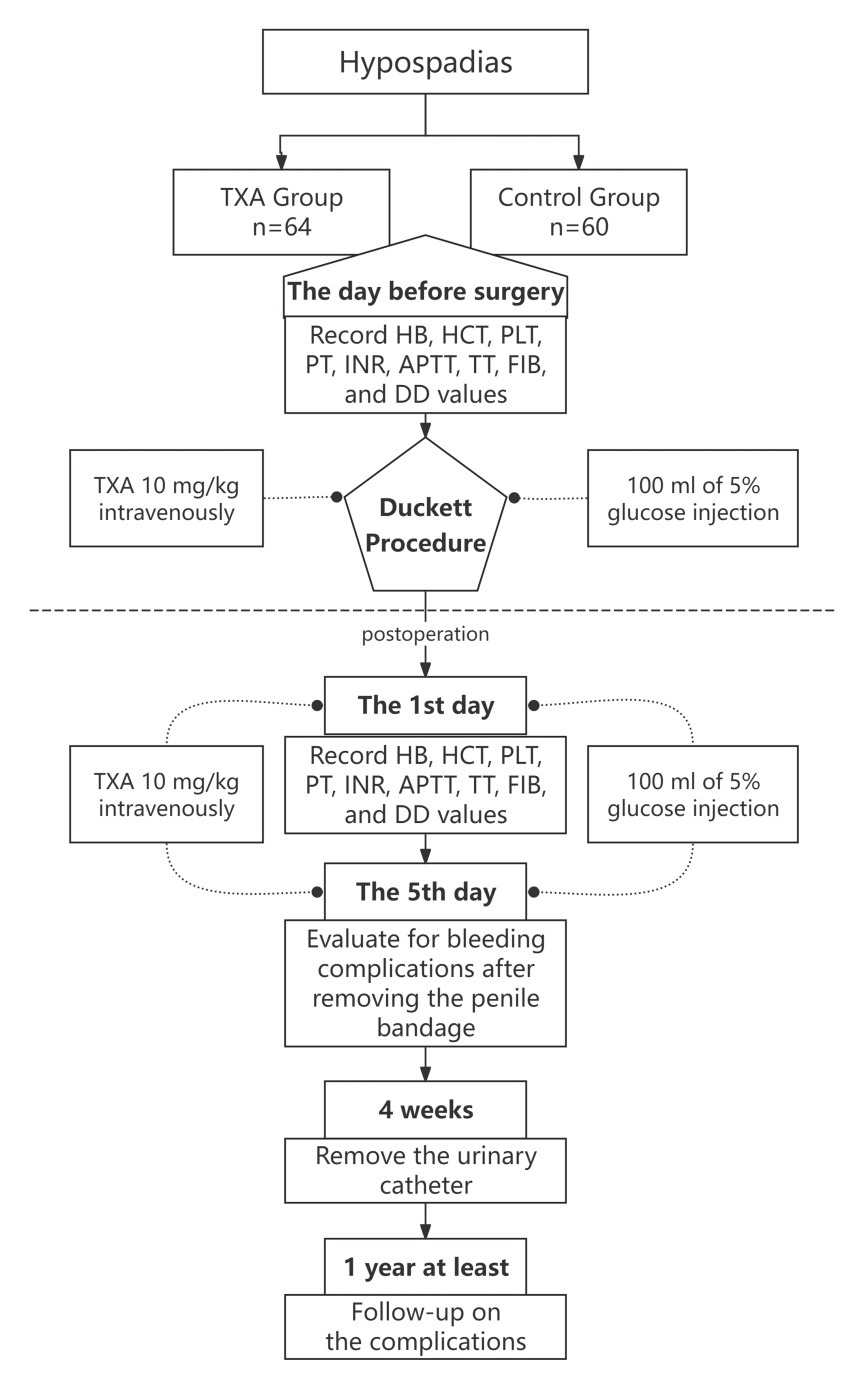
Consort diagram showing the study design TXA: tranexamic acid, HB: hemoglobin, HCT: hematocrit, PLT: platelet, PT: prothrombin time, INR: international normalized ratio, APTT: activated partial thromboplastin time, TT: thrombin time, FIB: fibrinogen, DD: D-dimer

Follow-up for at least 12 months postoperatively showed no complications (including urethral fistula, urethral stricture, urethral diverticulum, and recurrence of chordee), defined as the absence of complications (Figure 1).

Statistical methods

In our study, we hypothesized that TXA would reduce perioperative bleeding and postoperative complications. As a prospective study, we ensured that all surgical data were complete with no missing data. All statistical tests were two-sided. We utilized independent samples t-tests for continuous variables, chi-square tests or Fisher’s exact tests for categorical data, and logistic regression for multivariate analysis, all conducted using SPSS Statistics version 22.0 (IBM Corp. Released 2013. IBM SPSS Statistics for Windows, Version 22.0. Armonk, NY: IBM Corp.). Additionally, we generated receiver operating characteristic (ROC) curves using the pROC package version 1.18.5 (Comprehensive R Archive Network, https://cran.r-project.org/) in R version 4.3.2 (R Foundation for Statistical Computing, Vienna, Austria: https://www.R-project.org/) to assess the diagnostic performance of our primary outcome. In the revised manuscript, we will elaborate on how we tested assumptions for these analyses and clarify our approach to sensitivity analyses. A significance level of p<0.05 was considered statistically significant.

## Results

According to inclusion and exclusion criteria, a total of 124 patients with hypospadias underwent the Duckett procedure and completed follow-up: 64 in the TXA group and 60 in the control group. The follow-up duration for patients without complications was 28.21 ± 6.32 months in the TXA group and 28.95 ± 7.22 months in the control group. There was no statistically significant difference between the two groups (t=0.459, p=0.647). There were no statistically significant differences between the two groups in clinical data such as age, weight, length of hospital stay, duration of surgery, type of hypospadias, length of urethral defect, presence of chordee, use of DP, and incidence of complications (p>0.05, Table 1).

**Table 1:** Comparison of clinical data between the control group and the TXA group DP: dorsal plication, TXA: tranexamic acid

	Control n=60	TXA n=64	T/χ2	p-value
Age (years)	4.01 ± 3.35	4.30 ± 3.31	-0.484	0.629
Weight (kg)	20.82 ± 11.50	21.44 ± 11.18	-0.305	0.761
Length of hospital stay (d)	8.33 ± 1.68	8.89 ± 1.89	-1.731	0.086
Duration of surgery (min)	130.70 ± 33.39	122.67 ± 39.64	1.216	0.226
Type	-	-	0.011	0.915
Distal	0 (0)	0 (0)	-	-
Midshaft	36 (60.00)	39 (60.94)	-	-
Proximal	24 (40.00)	25 (39.06)	-	-
Length of the urethral defect (cm, x±s)	3.45 ± 0.84	3.57 ± 0.87	-0.779	0.437
Chordee＞30°	42 (70.00)	49 (76.56)	0.683	0.409
DP	52 (86.67)	56 (87.50)	0.019	0.890
Complications	27 (45.00)	28 (43.75)	0.020	0.889
Urethral fistula	19 (31.67)	18 (28.13)	0.696	0.952
Urethral stricture	4 (6.67)	4 (6.25)	-	-
Urethral diverticulum	2 (3.33)	4 (6.25)	-	-
Recurrence of chordee	2 (3.33)	2 (3.13)	-	-
Follow-up time	28.95 ± 7.22	28.21 ± 6.32	0.459	0.648

Table 2 showed pre- and postoperative differences in blood loss and coagulation-related indicators, as well as bleeding after removing the bandage. In the TXA group, intraoperative blood loss ((11.66 ± 5.14) ml vs. (13.55 ± 5.39) ml, t=1.998, p=0.048) was significantly lower compared to the control group. The number of electrotome usages ((12.41 ± 3.95) times vs. (14.58 ± 3.78) times, t=3.132, p=0.002) was significantly lower in the TXA group compared to the control group. ΔHB ((7.84 ± 6.62) g/L vs. (10.33 ± 6.97) g/L, t=2.041, p=0.043) and ΔHCT ((1.90 ± 1.56)% vs. (2.80 ± 2.04)%, t=2.783, p=0.006) were both significantly lower in the TXA group compared to the control group (p<0.05). There were no statistically significant differences in coagulation-related indicators (p>0.05).

**Table 2:** Comparison of blood loss and coagulation function between the control group and the TXA group HB: hemoglobin, HCT: hematocrit, PLT: platelet, PT: prothrombin time, INR: international normalized ratio, APTT: activated partial thromboplastin time, TT: thrombin time, FIB: fibrinogen, DD: D-dimer

	Control n=60	TXA n=64	T/χ2	p-value
Intraoperative blood loss (ml)	13.55 ± 5.39	11.66 ± 5.14	1.998	0.048
Number of electrotome usages (times)	14.58 ± 3.78	12.41 ± 3.95	3.132	0.002
ΔHB (g/L)	10.33 ± 6.97	7.84 ± 6.62	2.041	0.043
ΔHCT (%)	2.80 ± 2.04	1.90 ± 1.56	2.783	0.006
ΔPLT (10^9/L)	8.97 ± 70.48	28.63 ± 59.20	-1.686	0.094
ΔPT (s)	-0.22 ± 0.63	-0.04 ± 0.60	-1.680	0.096
ΔINR	-0.02 ± 0.06	-0.03 ± 0.06	0.555	0.580
ΔAPTT (s)	0.52 ± 2.53	1.39 ± 2.34	-1.984	0.050
ΔTT (s)	-0.03 ± 1.33	0.28 ± 1.50	-1.217	0.226
ΔFIB (g/L)	0.12 ± 0.50	0.26 ± 0.49	-1.568	0.120
ΔDD (mg/L)	-0.62 ± 0.64	-0.59 ± 0.72	-0.251	0.802
Bleeding after removing the bandage	12 (20.00)	9 (14.06)	0.776	0.378
Mild	9 (15.00)	6 (9.38)	0.948	0.814
Moderate	2 (3.33)	2 (3.13)	-	-
Severe	1 (1.67)	1 (1.56)	-	-

To further explore the impact of perioperative TXA application and blood loss on complications following the hypospadias Duckett procedure, we conducted a univariate analysis of complications. The results showed that urethral defect length, electrotome usage, intraoperative blood loss, ΔHB, and bleeding after removing the bandage were risk factors for complications (p<0.05, Table 3).

**Table 3:** Univariate analysis of postoperative complications occurring following the Duckett procedure DP: dorsal plication, TXA: tranexamic acid, HB: hemoglobin, HCT: hematocrit

	Non-complication n=69	Complication n=55	95% CI	p-value
Age (years)	4.32 ± 3.60	3.97 ± 2.95	0.868-1.078	0.550
Weight (kg)	21.36 ± 11.91	20.85 ± 10.57	0.965-1.028	0.803
Length of hospital stay (d)	8.65 ± 1.56	8.58 ± 2.09	0.803-1.192	0.829
Duration of surgery (min)	129.45±36.34	122.93 ± 37.43	0.985-1.005	0.327
Length of urethral defect (cm)	3.34 ± 0.89	3.73 ± 0.77	1.128-2.694	0.012
Chordee＞30°	48 (69.57)	43 (78.18)	0.690-3.560	0.283
DP	58 (84.06)	50 (90.91)	0.617-5.829	0.264
Type	-	-	0.865-3.715	0.116
Distal	0 (0)	0 (0)	-	-
Midshaft	46 (66.67)	29 (52.73)	-	-
Proximal	23 (33.33)	26 (47.27)	-	-
TXA	36 (52.17)	28 (50.91)	0.468-1.931	0.889
The number of electrotome usages (times)	12.55 ± 3.47	14.60 ± 4.36	1.040-1.260	0.006
Intraoperative blood loss (ml)	10.76 ± 4.29	14.85 ± 5.66	1.087-1.274	＜0.001
ΔHB (g/L)	7.38 ± 5.72	11.15 ± 7.64	1.029-1.158	0.004
ΔHCT (%)	2.13 ± 1.70	2.59 ± 2.02	0.940-1.390	0.180
Bleeding after removing the bandage	7 (10.14)	14 (25.45)	1.125-8.134	0.028
Mild	7 (10.14)	8 (14.55)	-	-
Moderate	0 (0)	4 (7.27)	-	-
Severe	0 (0)	2 (3.64)	-	-

Multiple logistic regression analyses included TXA application, urethral defect length, chordee >30°, classification, electrotome usage, intraoperative blood loss, ΔHB, and bleeding after removing the bandage. Surprisingly, although TXA was not identified as a factor influencing complications in univariate analysis, it emerged as a protective factor against complications in logistic regression analysis (p=0.049, OR=2.810, 95% CI: 1.004-7.868). Additionally, electrotome usage (OR=1.212, 95% CI: 1.067-1.377), intraoperative blood loss (OR=1.225, 95% CI: 1.101-1.362), and ΔHB (OR=1.153, 95% CI: 1.059-1.257) were independent risk factors for complications following the Duckett procedure (p<0.05, Table 4).

**Table 4:** Logistic regression analysis of postoperative complications occurring following the Duckett procedure TXA: tranexamic acid, HB: hemoglobin

	B-value	SE-value	Wald X2-value	p-value	OR	95% CI
TXA	1.033	0.525	3.869	0.049	2.810	1.004-7.868
Length of urethral defect (cm)	0.461	0.290	2.528	0.112	1.586	0.898-2.802
chordee＞30°	0.381	0.526	0.524	0.469	1.464	0.522-4.106
Type	-0.027	0.493	0.003	0.957	0.974	0.370-2.561
The number of electrotome usages (times)	0.192	0.065	8.727	0.003	1.212	1.067-1.377
Intraoperative blood loss (ml)	0.203	0.054	14.031	＜0.001	1.225	1.101-1.362
ΔHB (g/L)	0.143	0.044	10.640	0.001	1.153	1.059-1.257
Bleeding after removing the bandage	0.841	0.634	1.762	0.184	2.319	0.670-8.026
Constant	-9.193	2.054	20.021	＜0.001	＜0.001	-

**Figure 2:**
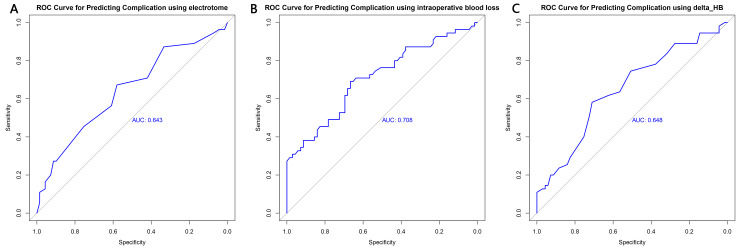
ROC curve of postoperative complications in the Duckett procedure A: number of electrotome usage, B: intraoperative blood loss, C: △HB, predicted the ROC curve of postoperative complications in the Duckett procedure ROC: receiver operating characteristic, HB: hemoglobin

We constructed ROC curves for electrotome usage, intraoperative blood loss, and ΔHB to predict complications following the Duckett procedure. According to the ROC curve for electrotome usage (AUC=0.643, Figure 2A), a Youden index maximum of 1.252 was calculated (sensitivity=0.673, specificity=0.580), with the best threshold of 12.5 times. Based on the ROC curve for intraoperative blood loss (AUC=0.708, Figure 2B), a Youden index maximum of 1.358 was calculated (sensitivity=0.691, specificity=0.667), with the best threshold of 12.3 ml. According to the ROC curve for ΔHB (AUC=0.648, Figure 2C), a Youden index maximum of 1.292 was calculated (sensitivity=0.582, specificity=0.710), with the best threshold of 9.5 g/L.

## Discussion

In recent years, the incidence of hypospadias has been increasing steadily, with an average reported incidence of approximately 2 per 1000, and it is increasing annually [7,8]. There are numerous surgical techniques for hypospadias repair, with a high overall incidence of complications, and no single technique has demonstrated absolute superiority [9]. The penis has a rich blood supply, and bleeding during hypospadias surgery is inevitable. Controlling bleeding during surgery can improve the surgeon's satisfaction with the procedure and reduce operating time [3]. The impact of the same amount of bleeding on the physiological status of children is significantly greater than in adults [10], highlighting the importance of controlling perioperative bleeding in pediatric patients. This study's results indicate that intraoperative bleeding volume and △HB are independent influencing factors for postoperative complications in the Duckett procedure. This is related to the increased risks of infection, local ischemia, and poor wound healing associated with bleeding. Moreover, multiple uses of diathermy due to increased intraoperative bleeding may increase local tissue damage. From a short-term perspective, although TXA can reduce intraoperative blood loss, its application does not have a significant impact on the length of hospital stay or surgery duration, and no patients in either group required transfusions postoperatively. However, multivariate analysis indicates that TXA can reduce the occurrence of postoperative complications, reflecting the potential significance of reducing intraoperative blood loss in improving patients' long-term recovery quality, which necessitates a long-term and comprehensive assessment of patients.

Tourniquets, local vasoconstrictors, and electrotome cautery are commonly used methods in clinical practice to control bleeding during hypospadias surgery. During surgery, methods such as rolling the edge of a rubber glove, latex tubing, etc. can be used as tourniquets [11]. Injection along the incision with adrenaline at concentrations of 1/100,000 to 1/200,000 can constrict blood vessels [12]. Both methods aim to reduce tissue perfusion for hemostasis. Shirazi et al. [13] found through animal experiments that local application of adrenaline for hemostasis does not increase adverse events. Alizadeh et al. [14] also confirmed that there is no statistically significant difference in complications between tourniquets and adrenaline for hemostasis, and adrenaline was found to be more effective. However, some question the safety of these methods, suggesting that changes in penile blood perfusion can affect tissue vitality and healing capacity. Kajbafzadeh et al. [4], through electron microscopy, observed that continuous or intermittent use of tourniquets for 30 minutes can cause reversible damage to the ultrastructure of the urinary epithelium. Gulburun et al. [15] confirmed through animal studies that restoration of blood flow after tourniquet release can cause I/R injury to tissues. The electrotome effect of diathermy can dissolve and denature collagen and FIB in tissue blood vessels, thereby occluding the vessel lumen to achieve hemostasis, but it can also cause thermal damage to tissues [16]. Caution should be exercised when using electrotome for hemostasis in penile surgery. For hypospadias surgery, the European Association of Urology recommends bipolar diathermy for cautery hemostasis [17]. Bipolar diathermy concentrates energy locally, preventing the spread of thermal damage, and it can safely achieve hemostasis in hypospadias surgery, reducing the need for tourniquets and adrenaline [18]. In this study, the complication group had significantly more electrotome usage than the non-complication group, which also demonstrates that the local thermal damage from electrotome increases postoperative complications.

Due to the safety concerns regarding hemostasis methods in hypospadias surgery mentioned above, this study explores the effect of intravenous TXA on blood loss in the Duckett procedure for hypospadias. TXA has been proven to reduce bleeding in orthopedic, cardiac, neurological, and obstetric surgeries, with a low incidence of adverse events such as thrombosis [19]. TXA is a derivative of lysine that competitively inhibits the lysine binding site on plasminogen molecules, preventing plasmin activation, stabilizing fibrin clots, inhibiting their degradation, and promoting hemostasis [20]. In recent years, further research into TXA has revealed numerous other properties beyond hemostasis. Reichel et al. [6] found through animal experiments that TXA can interfere with inflammatory cascades after I/R injury, effectively preventing neutrophil responses and vascular wall remodeling post-ischemia. Fallah et al. [21] discovered that TXA can delay the onset and reduce the severity of radiation dermatitis in mice by inhibiting plasminogen activation. Draxler et al. [22], through volunteer and cardiac surgery patient assessments, found that TXA modulates immune responses and reduces postoperative infection rates. In a study on joint replacement revisions, TXA was found to reduce the incidence of periprosthetic infections [23]. Sağlam et al. [24] found in an animal study simulating wound healing that local and intravenous TXA positively affected early wound healing. Given that complications of the Duckett procedure for hypospadias are also related to factors such as wound healing, infection, inflammatory responses, and I/R injury [25], we applied TXA for the first time during the perioperative period of pediatric hypospadias, with results showing that TXA not only reduces bleeding but also independently lowers the occurrence of post-hypospadias surgery complications. We also analyzed patients undergoing tubularized incised plate (TIP) urethroplasty during the same period and found that TXA did not have a similar effect on patients undergoing TIP procedures for hypospadias, possibly due to its protective effect against I/R injury to island flaps and the resulting inflammatory response (Tables 5-6).

**Table 5:** Univariate analysis of postoperative complications occurring following TIP surgery DP: dorsal plication, TXA: tranexamic acid, HB: hemoglobin, HCT: hematocrit, TIP: tubularized incised plate

	Non-complication n=103	Complication n=31	t/χ2	p-value
Age (years)	4.46 ± 3.48	5.14 ± 3.33	-0.967	0.335
Weight (kg)	21.34 ± 11.84	23.03 ± 10.46	-0.716	0.475
Length of hospital stay (d)	8.80 ± 2.48	8.65 ± 1.96	0.310	0.757
Duration of surgery (min)	97.50 ± 29.07	100.97 ± 31.30	-0.571	0.569
Length of urethral defect (cm)	1.74 ± 0.53	1.67 ± 0.34	0.617	0.538
Chordee＞30°	23 (22.33)	14 (45.16)	6.214	0.013
DP	29 (28.16)	14 (45.16)	3.162	0.075
Type	-	-	-	-
Distal	33 (32.04)	9 (29.03)	2.163	0.339
Midshaft	54 (52.43)	20 (64.52)	-	-
Proximal	16 (15.53)	2 (6.45)	-	-
TXA	60 (58.25)	13 (41.94)	2.558	0.110
The number of electrotome usages (times)	10.07 ± 3.13	13.03 ± 4.05	-4.311	＜0.001
Intraoperative blood loss (ml)	10.88 ± 4.13	13.40 ± 4.54	-2.915	0.004
ΔHB (g/L)	6.86 ± 7.24	10.90 ± 6.07	-2.819	0.006
ΔHCT (%)	1.83 ± 2.15	2.34 ± 2.17	-1.163	0.247
Bleeding after removing the bandage	13 (12.62)	7 (22.58)	1.861	0.172
Mild	11 (10.68)	6 (19.35)	-	-
Moderate	2 (1.94)	0 (0)	5.616	0.132
Severe	0 (0)	1 (3.23)	-	-

**Table 6:** Logistic regression analysis of postoperative complications occurring following TIP surgery TXA: tranexamic acid, HB: hemoglobin, TIP: tubularized incised plate

	B-value	SE-value	Wald X2-value	p-value	OR	95% CI
TXA	0.182	0.548	0.111	0.740	1.200	0.410-3.510
Length of urethral defect (cm)	-0.570	1.132	0.254	0.615	0.565	0.061-5.201
Chrodee＞30°	1.543	0.541	8.119	0.004	4.678	1.619-13.520
Type	-0.337	0.797	0.179	0.672	0.714	0.150-3.406
The number of electrotome usages (times)	0.290	0.079	13.486	＜0.001	1.337	1.145-1.561
Intraoperative blood loss (ml)	0.094	0.061	2.396	0.122	1.099	0.975-1.239
ΔHB (g/L)	0.096	0.038	6.361	0.012	1.100	1.022-1.185
Bleeding after removing the bandage	0.514	0.653	0.620	0.431	1.672	0.465-6.007
Constant	-5.678	1.651	11.833	0.001	0.003	-

Potential complications of TXA include thrombosis and seizures. Since TXA primarily inhibits fibrinolysis, theoretically it could induce thrombosis. A review involving 191 studies and 40,621 patients found that TXA use does not increase the occurrence of thromboembolic events [26]. However, studies also indicate that TXA use in trauma patients may increase the risk of venous thrombosis [27], suggesting the need to assess thromboembolic risk and closely monitor for thrombosis during clinical use of TXA. TXA can cross the blood-brain barrier and potentially induce seizures by antagonizing inhibitory γ-aminobutyric acid type A receptors in the brain [28]. Seizure occurrence may be closely related to the dosage of TXA. Sharma et al. [29] found in a multivariate analysis study of TXA use in cardiac surgery that patients receiving low-dose TXA (50 mg/kg) had a significantly lower risk of seizures compared to those receiving high-dose TXA (>80 mg/kg). However, another study on traumatic brain injury found that the risk of seizures in TXA-treated patients was similar to that in the control group [30]. In our study, patients with a history of epilepsy were excluded; no new cases of epilepsy occurred postoperatively; there were no significant differences in coagulation parameters between the TXA group and the control group; and there were no occurrences of thromboembolic events or other complications, indicating the safety of TXA application in pediatric hypospadias surgery.

This study also has certain limitations: there were variations in surgical experience among the surgeons performing hypospadias surgery, leading to individual differences in proficiency in penile anatomy dissection, management of unexpected situations during surgery, and handling of postoperative complications, thus introducing variability into the study. We conducted a combined analysis of all complications, but in reality, the factors contributing to each complication vary. The mechanisms by which TXA reduces complications are also unclear. Whether TXA plays a role in interfering with I/R injury, reducing inflammatory responses, and promoting healing in the Duckett procedure needs further verification through in vivo and in vitro experiments. The study focuses solely on TXA without comparing it to other hemostatic methods like tourniquets or local vasoconstrictors. In our future research, we will design experiments to compare TXA with other commonly used hemostatic methods to gain a more comprehensive understanding of their relative effectiveness and safety.

Another notable limitation of our study is the relatively short follow-up duration for assessing postoperative complications. While we included patients with a minimum follow-up of 12 months, the average follow-up time for both groups was approximately 28 months. This duration, while adequate for evaluating immediate and short-term outcomes, may not capture the full spectrum of potential long-term complications associated with TXA use. Future studies with extended follow-up periods are necessary to provide a more comprehensive understanding of TXA's effects on postoperative outcomes in pediatric hypospadias surgery.

## Conclusions

This study provides evidence that TXA plays a significant role in improving outcomes for pediatric hypospadias surgery performed using the Duckett procedure. By demonstrating that TXA effectively reduces intraoperative blood loss and ΔHB, our findings highlight its potential to lower the risk of postoperative complications, offering a practical approach to enhancing patient safety and surgical success. The observed benefits of TXA extend beyond mere hemostatic control, suggesting its role in mitigating adverse outcomes and enhancing overall recovery. However, the underlying mechanisms driving these benefits remain fully elucidated. Future research should aim to explore these mechanisms in greater depth to optimize TXA use and further improve surgical outcomes for pediatric patients undergoing hypospadias repair.
